# Opposing biological functions of the cytoplasm and nucleus DAXX modified by SUMO-2/3 in gastric cancer

**DOI:** 10.1038/s41419-020-2718-3

**Published:** 2020-07-08

**Authors:** Chenbin Chen, Xiangwei Sun, Wangkai Xie, Sian Chen, Yuanbo Hu, Dong Xing, Jianfeng Xu, Xiaodong Chen, Zhiguang Zhao, Zheng Han, Xiangyang Xue, Xian Shen, Kezhi Lin

**Affiliations:** 1 Department of Gastrointestinal Surgery, The Second Affiliated Hospital & Yuying Children’s Hospital of Wenzhou Medical University, Wenzhou, Zhejiang Province P.R. China; 2 Department of Microbiology and Immunology, Institute of Molecular Virology and Immunology, School of Basic Medical Sciences, Wenzhou Medical University, Wenzhou, Zhejiang Province P.R. China; 3 Department of Pathology, the Second Affiliated Hospital & Yuying Children’s Hospital of Wenzhou Medical University, Wenzhou, Zhejiang Province P.R. China; 4 Morphology Laboratory, School of Basic Medical Sciences, Wenzhou Medical University, Wenzhou, Zhejiang Province P.R. China

**Keywords:** Gastric cancer, Sumoylation

## Abstract

Death domain-associated protein (DAXX) is a complex biological multifunctional protein and is involved in the tumorigenesis and progression of multiple cancers. The accumulation of DAXX in the nucleus is a common phenomenon in tumor cells. However, altering the subcellular localizations of DAXX results in different biological functions, and we also found that its nuclear/cytoplasmic ratio (NCR) was associated with poor prognosis in gastric cancer (GC). In this study, we investigated the effect of cytoplasmic and nuclear DAXX (cDAXX and nDAXX) in GC and the underlying mechanisms. Immunohistochemical detection performed in 323 GC tissues reveled that cDAXX was associated with a better survival, while high nDAXX expression suggested a poorer prognosis outcome. Upregulation of DAXX in the cytoplasm inhibited cell proliferation and promoted apoptosis, whereas downregulation of DAXX in the nucleus displayed opposite effects. Moreover, Transwell assays revealed that DAXX enhanced GC cell migration and invasion. Analysis from the Gene Expression Profile Interactive Analysis (GEPIA) database showed that the expression of DAXX was significantly associated with SUMO-2/3 in GC tissues. Co-immunoprecipitation combined with immunofluorescence analysis indicated that DAXX interacted directly with SUMO-2/3. Subsequently, down-regulating the expression of SUMO-2/3 resulted in altered subcellular localization of DAXX. Bioinformatics analysis showed that RanBP2 may act as SUMO E3 ligase to promote nuclear-plasma transport via combining with RanGAP1. Taken together, our results indicated that DAXX plays opposing roles in GC and suggest a new model whereby cDAXX, nDAXX, and SUMO-2/3 form a molecular network that regulates the subcellular localization of DAXX and thereby modulates its opposing biological effects. Thus, our findings provide a foundation for future studies of DAXX as a novel therapeutic target for patients with GC.

## Introduction

Gastric cancer (GC) is the fourth most common malignant disease worldwide and the third greatest contributor to cancer-related death^1^, particularly in China^2^. Despite multiple advances in diagnostic tools and surgical techniques, the 5-year survival rate for patients with GC is still <10%^3^ because most patients are diagnosed with advanced or metastatic stage cancer. Hence, it is necessary to identify more specific biomarkers to find new therapeutic strategies for patients with GC.

Death domain-associated protein (DAXX), also known as death-associated protein 6 (DAP6), was originally identified as a novel FAS-interacting protein^4^ that enhances FAS-inducible and transforming growth factor-β-dependent cellular apoptosis. DAXX has also been reported to play roles in a variety of biological functions, including mediating apoptosis^5,6^, participating in tumorigenesis^7,8^, regulating transcriptional processes^9,10^, and even preventing viral invasion^11,12^. Previous studies have shown that DAXX can be localized to the promyelocytic leukemia protein nucleus (PML), nucleoplasm, nucleoli, cytoplasm, and heterochromatin, and alters its subcellular localization by modifying or interacting with other proteins, thereby regulating the corresponding downstream pathway and exerting different biological effects^13–15^. Our previous study found that DAXX expression was elevated in GC tissues and that DAXX displayed a high NCR in tumor tissues^16^. Clinical data has also revealed that the DAXX NCR is closely related to the malignant phenotype of GC; however, the corresponding role of DAXX in different subcellular localizations in GC cells remain poorly defined.

Post-translational protein modifications by the small ubiquitin-like modifier (SUMO) (SUMOylation), a highly dynamic reversible process that primarily acts on transcription factors, have been increasingly recognized to play an important role in protein function^17^. Previous studies have shown that DAXX, a transcriptional co-repressor, has two independent SUMO interaction motifs (SIMs) located at the N- and C-termini^18^. SUMO protein family consists of proteins of 98 amino acids, similar to ubiquitin. There are four SUMO isomers, namely SUMO-1, SUMO-2, SUMO-3, and SUMO-4^19^. Studies have shown that SUMO-1 mainly modifies proteins under physiological conditions, whereas SUMO-2/3 are two highly similar isoforms that exist in free form in cells under most normal conditions and switch to a conjugated form in response to cellular stresses. The majority of studies have focused on the effects and mechanisms via which DAXX and SUMO-1 interact^20,21^; however, few have examined changes in DAXX and SUMO-2/3 during tumor development.

In this study, we investigated the expression and prognostic value of DAXX using TCGA datasets and examined cDAXX and nDAXX expression in GC tissues by immunohistochemistry (IHC). We also investigated the effect of DAXX subcellular localization on proliferation, invasion, and migration by overexpressing and silencing DAXX in GC cells and examined the interaction between DAXX and SUMO-2/3 and its effect on subcellular localization. The possible involvement of SUMO E3 ligases was analyzed using bioinformatics. Together, our data demonstrate that DAXX may be a novel therapeutic target for the treatment of patients with GC.

## Materials and methods

### Patients and tissue samples

We retrospectively reviewed patients who underwent surgical resection for GC from December 2008 to July 2011 at the Second Affiliated Hospital of Wenzhou Medical University (Wenzhou, People’s Republic of China). A total of 343 patients were included in this study and each patient was followed up to collect pathology reports and conditions. None of the patients received radiotherapy or chemotherapy before surgery. All 343 GC tissues and 20 paired adjacent normal tissues (at least 10 cm from the negative margin) were fixed in formalin, embedded in paraffin, and histologically diagnosed according to World Health Organization (WHO) criteria and American Joint Committee on Cancer (AJCC)/Union for International Cancer Control (UICC) TNM classification system. At the same time, clinical pathological characteristics such as gender, age, tumor size, depth of invasion, lymph node metastasis, TNM stage, Lauren type, serum carcinoembryonic antigen (CEA), carbohydrate antigen 19-9 (CA 19-9), and carbohydrate antigen 72-4 (CA 72-4) levels were collected for each patient. This study was approved by the Ethics Committee of Wenzhou Medical University (2019046). Written informed consent was obtained from all study participants.

### Immunohistochemistry

DAXX expression was detected in tumor tissue and normal tissue using IHC. Tissue microarrays (TMAs) were constructed and IHC was performed on the TMA slides as described previously^22^. Briefly, the slides were incubated with mouse anti-DAXX antibodies (1:200 dilution, Santa Cruz Biotechnology, Santa Cruz, Calif., USA; sc-8043), with antibody diluent buffer used as a negative control. The slides were then stained with DAB (Dako, Cytomation, Calif., USA), counterstained with hematoxylin, and evaluated independently by two blinded pathologists according to the following scoring criteria: 0 (negative), 1 (weak), 2 (middle), or 3 (strong). The degree of staining was scored according to the proportion of the positive staining area relative to the whole cancerous area, as follows: 0 (<5%), 1 (5–25%), 2 (26–50%), 3 (51–75%), and 4 (>75%). The total score was obtained by multiplying the percentage score by the stain intensity score and inconsistencies between the reviewers’ scores were reviewed until a consensus was reached. The DAXX staining of 20 pairs of GC and adjacent tissues was also examined using Image-Pro-Plus (version 6.0, Media Cybernetics, Rockville, USA) to verify our results. Receiver operating characteristic (ROC) curve analysis was performed to select a cut-off score.

### Cell culture

The human GC cell lines AGS, BGC-823, MGC-7901, SGC-7901, and MKN-45, and the normal human immortalized gastric mucosal cell line GES-1 were obtained from the cell bank of the Chinese Academy of Medical Sciences (Shanghai, China). The cells were cultured in RPMI-1640 (Gibco, Thermo Fisher Scientific) or Dulbecco’s Modified Eagle Medium (DMEM; Gibco, Thermo Fisher Scientific) supplemented with 10% fetal bovine serum (Gibco) at 37 °C in a humidified atmosphere with 5% CO_2_. The medium was changed every 2 days and cells were collected in logarithmic growth phase for further experiments.

### Plasmid and cell transfection

DAXX cDNA was cloned into pcDNA3.1 vector. For transient small interfering RNA (siRNA) transfection, DAXX siRNA (si-DAXX-1, si-DAXX-2, and si-DAXX-3), SUMO-2/3 siRNA (si-SUMO-2-1, si-SUMO-2-2, si-SUMO-3-1, and si-SUMO-3-2), and a negative control (si-NC) were synthesized by RiboBio (Guangzhou, China; Table 1). According to the manufacturer’s instructions, plasmid and siRNAs were transfected into GC cells at 60–70% confluency using Lipofectamine 2000 reagent (Invitrogen Life Technologies^®^, Carlsbad, Calif., USA) and incubated at 37 °C before being harvested.

**Table 1:** Sequences of siRNA.

Name	Sense (5′-3′)
siDAXX-1	CGTTGACCCTGCACTATCA
siDAXX-2	CAGCCAAGCTCTATGTCTA
siDAXX-3	GGATCAGGGCCATTAGGAA
siSUMO-2-1 (3′-UTR)	TGATACTGATGCCAAACAA
siSUMO-2-2 (3′-UTR)	GGTCACTACAGTCTTTATT
siSUMO-3-1	CAGAGAATGACCACATCAA
siSUMO-3-2	GGCAGATCAGATTCAGGTT

### RNA isolation and quantitative real-time polymerase chain reaction

Total RNA was extracted from cells using TRIzol reagent (Invitrogen Life Technologies^®^) according to the manufacturer’s instructions and was then reverse transcribed and first-strand cDNA was reverse transcribed using a ReverTraAce^®^ qPCR RT Kit (Toyobo^®^, Tokyo, Japan). To detect relative DAXX and SUMO-2/3 mRNA levels, quantitative real-time polymerase chain reaction (qRT-PCR) was performed using an SYBR^®^ Green PCR Kit (QIAGEN) with a Real-Time PCR Detection System (BioRad, CFX96). All tests were repeated independently three times. Relative mRNA levels were normalized to endogenous GAPDH mRNA using the relative quantification comparative Ct method. The primer sequences are shown in Table 2.

**Table 2:** Sequences of primers used for qRT-PCR.

Gene (Homo)	Sequence (5′–3′)	
DAXX	Sense	TGACCCAGACTCCGCATAC
	Anti-sense	CCGAAGCACATCCCCATA
SUMO-2	Sense	ATGGCCGACGAAAAGCCCAAGGAAG
	Anti-sense	CTTCATCCTCCATTTCCA
SUMO-3	Sense	GAATGACCACATCAACCTGAAGG
	Anti-sense	GCCCGTCGAACCTGAATCT
GAPDH	Sense	CAGGGCTGCTTTTAACTCTGGTAA
	Anti-sense	GGGTGGAATCATATTGGAACATGT

### Preparation of separate nuclear and cytoplasmic lysates

The subcellular localization of DAXX was assessed by nuclear-cytoplasmic protein separation using an Ambion PARIS^TM^ kit (Thermo Fisher Scientific Inc., USA). Briefly, 1 × 10^7^ transfected cells were collected, washed once with cold phosphate-buffered saline (PBS), and placed on ice for 15 min. The cells were resuspended in 200 μL of ice-cold cell fractionation buffer, incubated on ice for 10 min and then centrifuged at 500 × *g* for 1–5 min at 4 °C before the cytoplasmic fraction was carefully absorbed. Nuclear proteins were collected after cell disruption buffer had cleaved the nuclear precipitate.

### Co-immunoprecipitation

Co-immunoprecipitation (Co-IP) assays were performed to assess the direct interaction between DAXX and SUMO-2/3 using a Pierce Magnetic HA-Tag IP/Co-IP Kit (Thermo Fisher Scientific Inc., USA) according to the manufacturer’s instructions. After the cells had been harvested, supernatants containing DAXX-HA-tagged proteins were added to pre-washed magnetic beads and incubated for 30 min at 25 °C. The beads were separated using a magnetic stand and the target protein resuspended in 100 μL of 2× loading buffer supplemented with 2.5 μL 2 M DTT for further experiments.

### Western blotting

Transfected cells were washed with PBS and lysed in RIPA buffer containing protease and phosphatase inhibitor cocktails. The supernatant was collected and preserved at –80 °C. Protein samples were separated by sodium dodecyl sulfate-polyacrylamide gel electrophoresis (SDS-PAGE), transferred to polyvinylidene fluoride (PVDF) membranes, and incubated with 5% skim milk for 1 h. The membranes were incubated overnight at 4 °C with the following specific primary antibodies: human DAXX (diluted 1:200), SUMO-2/3 (Cell Signaling Technology (CST), Danvers, MA, USA; #4971), α-Tubulin (CST; #3873), Lamin A/C (CST; #4777), HA-Tag (CST; #3724) (diluted 1:1000), and GAPDH (diluted 1:1000, Proteintech, Chicago, USA; 60004-1-Ig). The membranes were then incubated with secondary horseradish peroxidase (HRP)-conjugated antibodies (dilution 1:5000, Cell Signaling Technology) for 2 h at 25 °C. Proteins were then detected using enhanced chemiluminescence reagent and observed using a Biorad Imaging System (Biorad). The grayscale value represents the amount of target protein and was calculated by gray scanning using ImageJ software (NIH, Bethesda, USA). All protein expression levels were evaluated relative to GAPDH expression.

### Cell proliferation and colony formation assays

Cell proliferation was detected using a Cell Counting Kit-8 (CCK-8) assay. Briefly, cells were seeded onto 96-well plates (5 × 10^3^ cells/well; five replicates) and cultured for 24 h. After cell transfection for 24, 48, or 72 h, 10 μL/well of CCK8 solution (Solarbio Science & Technology Co., Ltd., Beijing, China) was added to each plate, incubated for 3 h at 37 °C, and absorbance measured at 450 nm using a micro-plate reader.

For the colony formation assays, transfected cells were cultured in six-well plates at a density of 1 × 10^3^ cells per well for 10 days. Subsequently, the cells were washed with PBS, fixed with 4% paraformaldehyde for 15 min, washed with PBS, and stained with 0.1% crystal violet (Beyotime Biotechnology, Shanghai, China) for 15 min. The number of colonies was then counted under a microscope.

### Transwell migration and invasion assays

Transwell assays were performed to assess cell migration and invasion. For the migration assay, 2 × 10^5^ transfected cells were suspended in serum-free DMEM (200 μL) and added to the uncoated upper Transwell chamber. For the invasion assay, cells were seeded into the upper chamber coated with 100 μL Matrigel (BD Pharmingen, San Jose, Calif., USA) diluted in serum-free DMEM (1:10). For both assays, DMEM containing 10% FBS (600 μL) was added to the lower chamber and, after incubation at 37 °C for 24 h, cells were fixed with 4% paraformaldehyde for 15 min, stained with 0.1% crystal violet for 15 min, and then imaged. Positive cells were counted using ImageJ software.

### Cell cycle and apoptosis assays

For cell cycle analysis, transfected cells were harvested, fixed with 75% ethanol for 2 h at −20 °C, and treated with propidium iodide (PI; 100 μg/mL; BD Pharmingen) and RNase A (10 μg/mL) for 15 min. Cell cycle distribution was analyzed by flow cytometry using a BD FACSCalibur flow cytometer (BD Biosciences, San Jose, CA, USA). Data were analyzed using Modfit software.

Cell apoptosis assays were used to analyze the percentage of apoptotic cells. According to the manufacturer’s instructions, transfected cells were detected using an Annexin-V-FITC Cell Apoptosis Kit (BD Pharmingen). Data were analyzed using Flowjo software.

### Immunofluorescence

Cells were seeded into a six-well plate containing glass coverslips and cultured at 37 °C for 24 h after transfection. The cells were then washed twice with PBS, fixed with 4% paraformaldehyde for 15 min at 25 °C, and permeabilized with 0.5% Triton-X100-PBS for 15 min. After blocking with PBST containing 5% goat serum for 30 min at 37 °C, the cells were incubated with primary anti-DAXX (mouse, Santa Cruz Biotechnology), anti-HA-Tag (rabbit, Cell Signaling Technology), anti-HA-Tag (mouse, Cell Signaling Technology), and anti-SUMO2/3 (rabbit, Cell Signaling Technology) antibodies at 4 °C overnight, and then secondary FITC-conjugated goat anti-rabbit, DyLight 594-conjugated goat anti-mouse, DyLight 549-conjugated goat anti-rabbit, and FITC-conjugated goat anti-mouse antibodies for 1 h. Cell nuclei were stained with 4′,6-diamidino-2-phenylindole (DAPI). Digital images were collected using a fluorescence microscope.

### Bioinformatics analysis

We used GEPIA (http://gepia.cancer-pku.cn/), an online tool developed by Peking University, to perform integrated analysis on the DAXX expression profiles of GC samples (*n* = 408) and normal gastric mucosa samples (*n* = 36) from the TCGA and GTEx databases. The prognostic value of DAXX expression in the GC cohort (*n* = 384) was evaluated using the Kaplan–Meier method and log-rank test, while correlations between DAXX and SUMO-1, 2, and 3 expression were analyzed using GEPIA and STRING (https://string-db.org/).

RNA-Sequencing gene expression data and clinical data of GC were downloaded from TCGA database (https://portal.gdc.cancer.gov/). To further determine the E3 ubiquitin ligase (E3) involved in DAXX SUMOylation, samples were divided into high and low expression groups according to the expression of DAXX, SUMO-2, and SUMO-3. The differentially expressed genes (DEGs) of these three groups were analyzed using the R edgeR package. Considering that E3 only acted as a bridge in the reaction and that the change in expression was not very large, we defined the significance criteria as the absolute value of log (fold-change) > 0 and the adjusted *P* value < 0.05. The Venn diagrams (http://bioinformatics.psb.ugent.be/webtools/Venn/) was used to obtain genes affected by the three genes. Next, we performed functional enrichment analysis with Gene Ontology (GO) terms and Kyoto Encyclopedia of Genes and Genomes (KEGG) pathways using the R clusterProfiler package to identify the relevant gene annotations and selected signature genes involved in the SUMOylation process. In addition, we analyzed these signature genes using STRING (https://string-db.org/) and correlations with SUMO-2/3.

### Statistical analysis

Statistical analyses were performed using Statistical Product and Service Solutions (SPSS, version 20.0, IBM, New York, USA) and GraphPad Prism 7.0 software. Continuous variables were expressed as the mean ± standard deviation (SD) and were analyzed by unpaired Student’s *t* tests for the comparison of two groups when the variances are equal. Categorical variables were analyzed using the *χ*^2^ test or Fisher’s exact test. Survival analyses were plotted using the Kaplan–Meier method and were compared using the log-rank test. *P < 0.05, **P < 0.01, and ***P < 0.001 indicated statistically significant differences.

## Results

### DAXX is highly expressed in GC and exerts opposite effects in different subcellular locations

Previously, we showed that DAXX is highly expressed in GC tissues and that the DAXX NCR is significantly higher in GC tissues than in adjacent normal tissues. To confirm these findings, we performed IHC analysis to detect DAXX expression in the tumor and normal tissues of 20 patients with GC (Supplementary Fig. 1A). Consistent with previous results, the DAXX positive rate was higher in tumor tissues (65%) than in adjacent normal tissues (50%; Supplementary Fig. 1B), while DAXX expression was significantly higher in the nucleus than in the cytoplasm (Supplementary Fig. 1C, D). These findings are also consistent with the data from the TCGA database (Supplementary Fig. 1E). Moreover, qRT-PCR and western blotting showed that DAXX expression was higher in GC cell lines (particularly BGC-823) than in the GES-1 (Supplementary Fig. 1G, H).

**Fig. 1:**
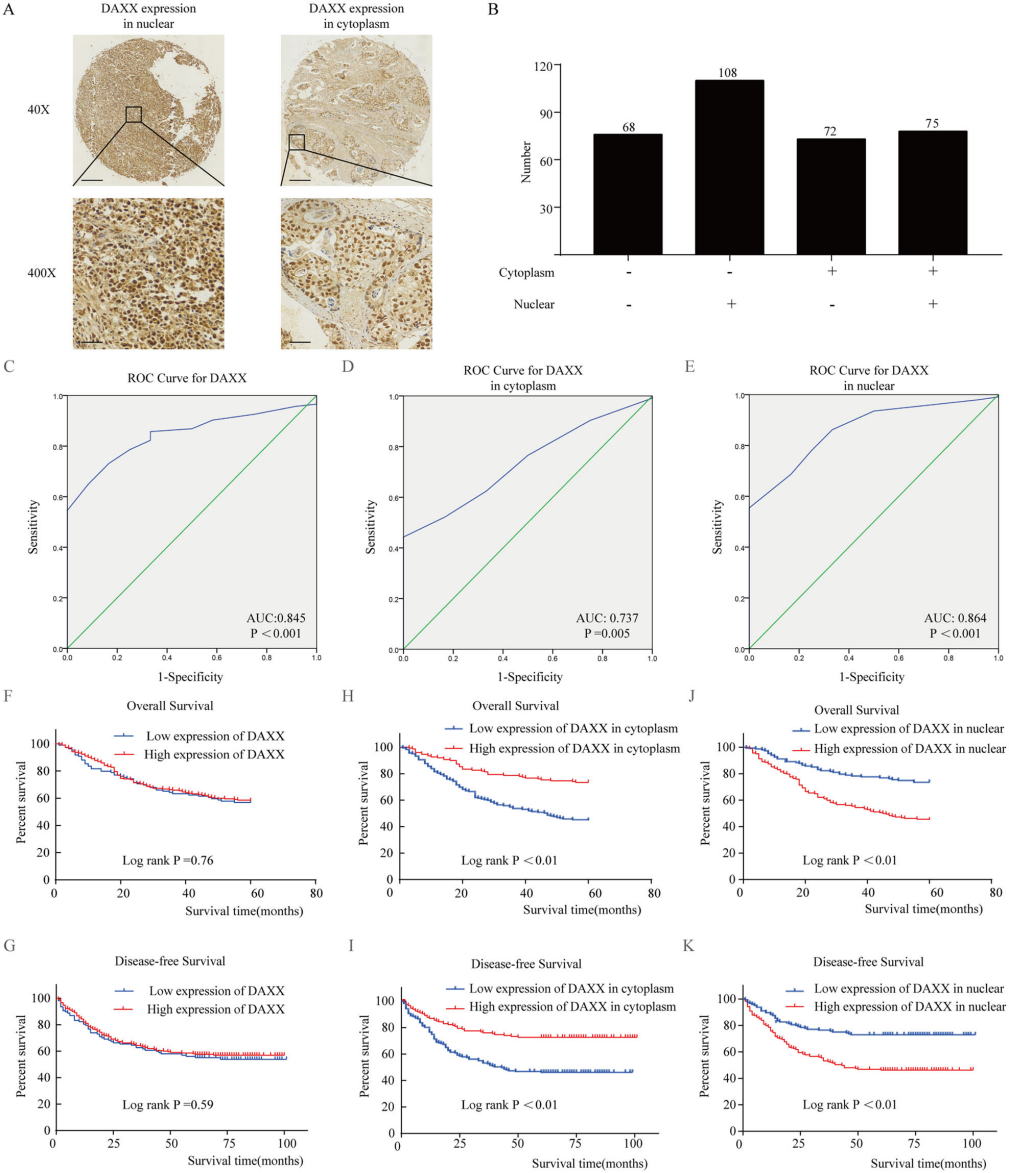
DAXX expression in the nucleus and cytoplasm have opposite effects in patients with gastric cancer (GC). **a** DAXX expression in the cytoplasm and nucleus, respectively. Brown represented the positive expression of DAXX protein, and blue represented the nucleus. And DAXX was mainly located in the nucleus of GC. (×40: Scale bar = 200 μm; ×400: Scale bar = 20 μm). **b** Number of GC patients with different DAXX expression levels. **c**–**e** ROC curves based on DAXX expression and its expression in the cytoplasm and nucleus. **f**, **g** The DAXX expression in GC patients was not associated with overall survival and disease-free survival. **h**, **i** Higher DAXX expression in the cytoplasm in GC patients had a longer overall survival and disease-free survival than those of lower expression patients. **j**, **k** Lower DAXX expression in the cytoplasm in GC patients had a longer overall survival and disease-free survival than those of higher expression patients.

Furthermore, we examined cDAXX and nDAXX expression in the tumor tissues of 323 patients with GC by IHC, with the results indicating that DAXX was mainly expressed in the nucleus of tumor tissues (Fig. 1a). We calculated total DAXX, cDAXX, and nDAXX expression, obtained corresponding cut-off values from the ROC curve (Fig. 1c–e), and thus divided the tumor tissues into a high expression group and low expression group (Fig. 1b). ROC curve analysis predicted that DAXX was able to classify GC tissues with high accuracy. Furthermore, we found that DAXX expression in different locations was significantly associated with lymph node metastasis, Lauren type, CA72-4 and relapse (*P* < 0.01; Table 3). Interestingly, DAXX expression in different subcellular locations also displayed opposite effects on the prognosis of patients with GC. Higher cDAXX expression in GC patients was associated with a longer overall survival and disease-free survival (*P* < 0.01; Fig. 1h, i), whereas high nDAXX expression suggested a poorer survival rate (*P* < 0.01). However, total DAXX expression was not associated with overall survival (*P* = 0.76) or disease-free survival (*P* = 0.59; Fig. 1f, g), consistent with the findings from the TCGA and GTEx databases (Supplementary Fig. 1F). Taken together, these results indicate that DAXX is overexpressed and associated with poor prognosis in GC depended on its different subcellular locations.

**Table 3:** The clinical characteristics of patients according to the expression of DAXX in cytoplasm and nuclear.

Variables	Expression of DAXX in cytoplasm		*P* value	Expression of DAXX in nuclear		*P* value
	High (*n* = 147)	Low (*n* = 176)		High (*n* = 183)	Low (*n* = 140)	
Gender, *n* (%)			0.412			0.188
Male	108	122		125	105	
Female	39	54		58	35	
Age (years), *n* (%)			0.917			0.727
<60	68	83		84	67	
≥60	78	93		99	73	
Tumor size (cm), *n* (%)			0.272			0.238
<4	75	79		82	72	
≥4	73	97		101	68	
Depth of invasion, *n* (%)			0.105			0.602
T1 + T2	56	52		59	49	
T3 + T4	91	124		124	91	
Lymph node metastasis, *n* (%)			<0.01^a^			0.561
N0 + N1	94	80		96	78	
N2 + N3	53	96		87	62	
M stage			0.599			0.680
M0	145	171		178	138	
M1	2	5		5	2	
TNM stage			0.003^a^			0.407
I + II	83	70		83	70	
III + IV	64	106		100	70	
Lauren type			<0.01^a^			0.540
Intestinal	85	61		80	66	
Diffuse	62	115		103	74	
CEA	8.40 ± 21.60	6.50 ± 21.83	0.449	8.26 ± 22.95	6.09 ± 19.78	0.394
CA199	36.64 ± 88.87	55.28 ± 159.17	0.219	53.43 ± 150.50	36.27 ± 95.15	0.286
CA724	7.22 ± 15.93	16.89 ± 47.37	0.047	13.69 ± 36.15	9.77 ± 36.98	0.465
Chemotherapy			0.261			0.173
No	45	44		45	44	
Yes	102	132		138	96	
Borrmann			0.438			0.203
I	18	13		14	17	
II	57	74		69	62	
III	62	73		83	52	
IV	10	16		17	9	
Relapse			<0.01^a^			<0.01^a^
No	105	74		81	98	
Yes	42	102		102	42	

*CEA* carcinoembryonic antigen, *CA199* carbohydrate antigen199, *CA724* carbohydrate antigen724, *TNM* tumor node and metastasis.

^a^Statistically significant (*P* < 0.01).

### DAXX alters GC cell proliferation by changing cDAXX and nDAXX expression

**Fig. 2:**
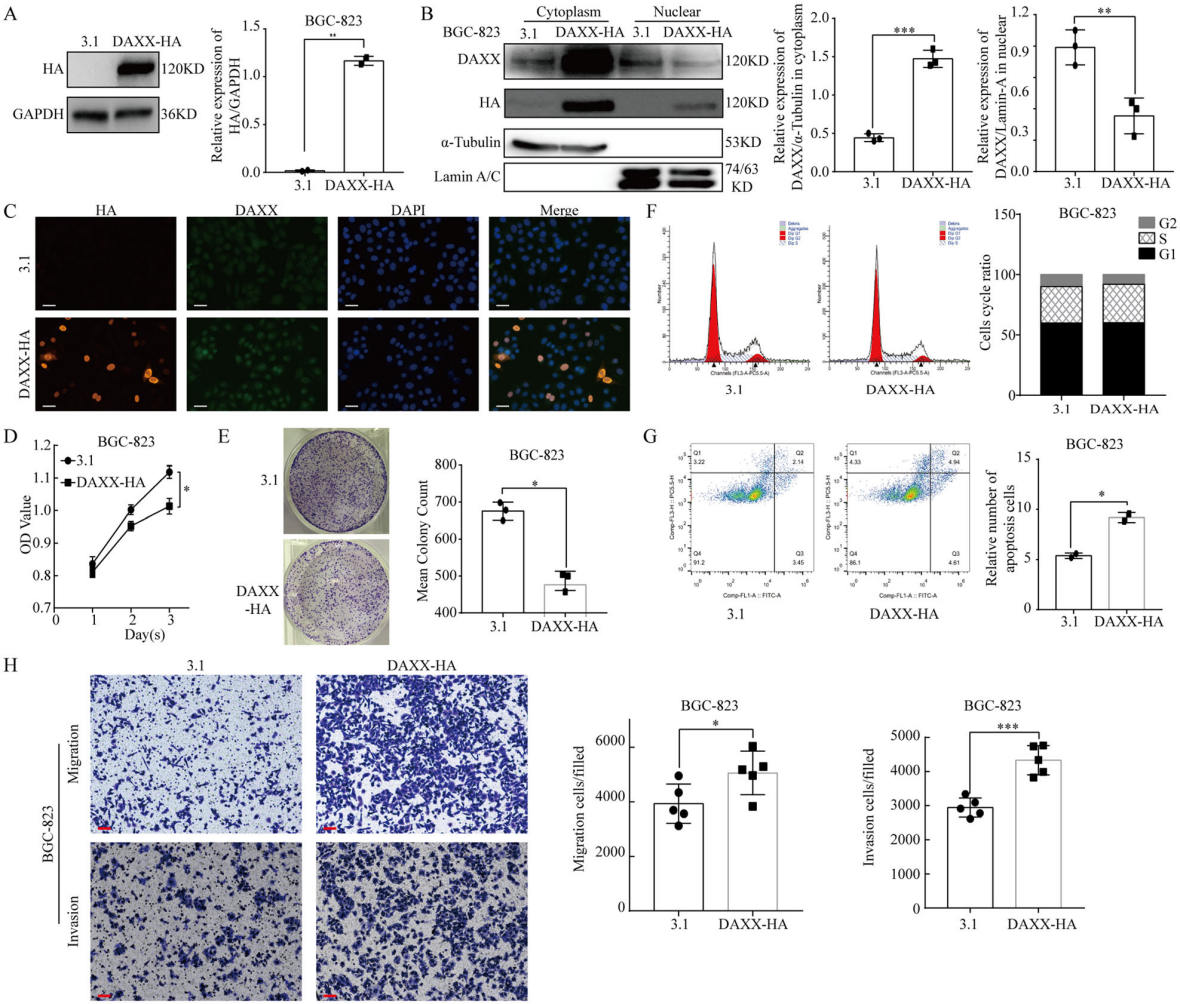
Increasing DAXX expression in the cytoplasm inhibits proliferation and promotes migration, and invasion of BGC-823 cells. **a** Western blot analysis of DAXX on BGC-823 transfected with DAXX-HA. **b**, **c** The expression of cDAXX was significantly increased when transfected with DAXX-HA. (Scale bar = 10 μm). **d**, **e** Increasing DAXX expression in the cytoplasm inhibits proliferation on BGC-823. **f** Cell cycle assays showed that increasing DAXX expression did not influence the cell cycle. **g** Apoptosis assays showed that increasing DAXX expression significantly promoted the apoptosis. **h** Overexpression of DAXX promoted migration and invasion on BGC-823. (Scale bar = 10 μm) *P < 0.05, **P < 0.01, and ***P < 0.001.

To study the biological effects of DAXX on gastric cancer cells, we constructed a DAXX-HA overexpression plasmid (Fig. 2a). Then we isolated cDAXX and nDAXX in the cells and used immunofluorescence (IF) assays to further detect their subcellular distribution. DAXX expression was significantly higher in the cytoplasm, although DAXX was still highly expressed in the nucleus after overexpression (Fig. 2b, c and Supplementary Fig. 2A). To examine the effect of DAXX on cell proliferation, we conducted CCK8 assays, finding that BGC-823 cell proliferation was significantly inhibited by DAXX overexpression compared to the control group (*P* < 0.05; Fig. 2d). Colony formation assays revealed that BGC-823 cell colony numbers were significantly lower after DAXX overexpression (*P* = 0.04; Fig. 2e); however, cell cycle assays detected no significant differences between the cell division cycle in the control and DAXX overexpression groups (Fig. 2f). Apoptosis assays using flow cytometry revealed that apoptosis was significantly promoted in the DAXX group compared with the control group (*P* = 0.02; Fig. 2g). Collectively, these findings suggest that DAXX may inhibit cell proliferation and induce apoptosis by increasing cDAXX expression.

**Fig. 3:**
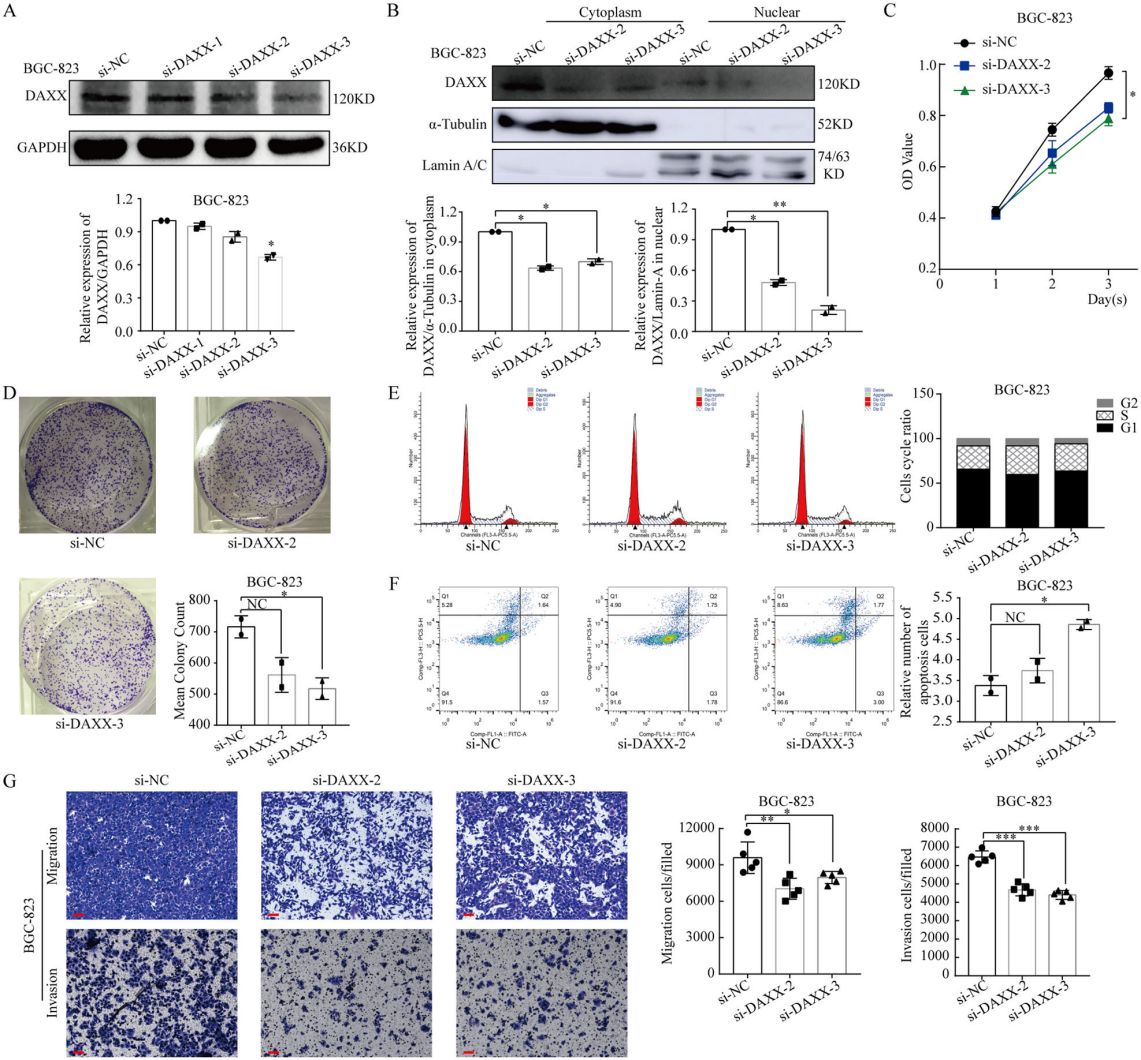
Decreasing DAXX expression in the nucleus inhibits proliferation, migration, and invasion of BGC-823 cells. **a** Western blot analysis of DAXX on BGC-823 transfected with si-DAXX. **b** The expression of DAXX was significantly decreased when transfected with si-DAXX, especially nDAXX. **c**, **d** Decreasing DAXX expression in the nucleus inhibits proliferation on BGC-823. **e** Cell cycle assays showed that decreasing DAXX expression did not influence the cell cycle. **f** Apoptosis assays showed that decreasing DAXX expression significantly promoted the apoptosis. **g** Knocking down of DAXX suppressed migration and invasion in BGC-823. (Scale bar = 10 μm) *P < 0.05, **P < 0.01, and ***P < 0.001.

At the same time, we also used siRNAs to knock down DAXX expression in BGC-823 cells (Fig. 3a). We then transfected the BGC-823 cells with DAXX siRNAs (si-DAXX-2 and si-DAXX-3) and a negative control (si-NC), with western blot assays revealing that silencing DAXX significantly decreased total DAXX expression and nDAXX expression in particular (Fig. 3b and Supplementary Fig. 2B). CCK-8 and colony formation assays both revealed that knocking down DAXX expression significantly inhibited cell proliferation (Fig. 3c,d); however, knocking down DAXX expression had no significant effect on the cell cycle (Fig. 3e). Apoptosis assays showed that silencing DAXX significantly increased the cell apoptosis ratio (Fig. 3f). Taken together, these findings indicate that DAXX can inhibit GC cell proliferation and oppose anti-apoptotic pathways by significantly decreasing their nDAXX expression.

### DAXX can significantly promote GC cell migration and invasion

To further explore the relationship between DAXX and GC development, we examined the effects of DAXX on GC cell migration and invasion by overexpressing or knocking down DAXX expression using DAXX recombinant plasmids and si-DAXX, respectively. As shown in Fig. 2h, the number of migrating and invading BGC-823 cells was significantly higher after transfection with the DAXX plasmid than in the control cells (*P* < 0.01). Conversely, knocking down DAXX significant decreased the ability of GC cells to migrate and invade (Fig. 3g). Thus, these results confirm that DAXX may affect the migration and invasion of GC cells via its nuclear expression.

### SUMO-2/3 modulates the subcellular localization of DAXX in GC cells

**Fig. 4:**
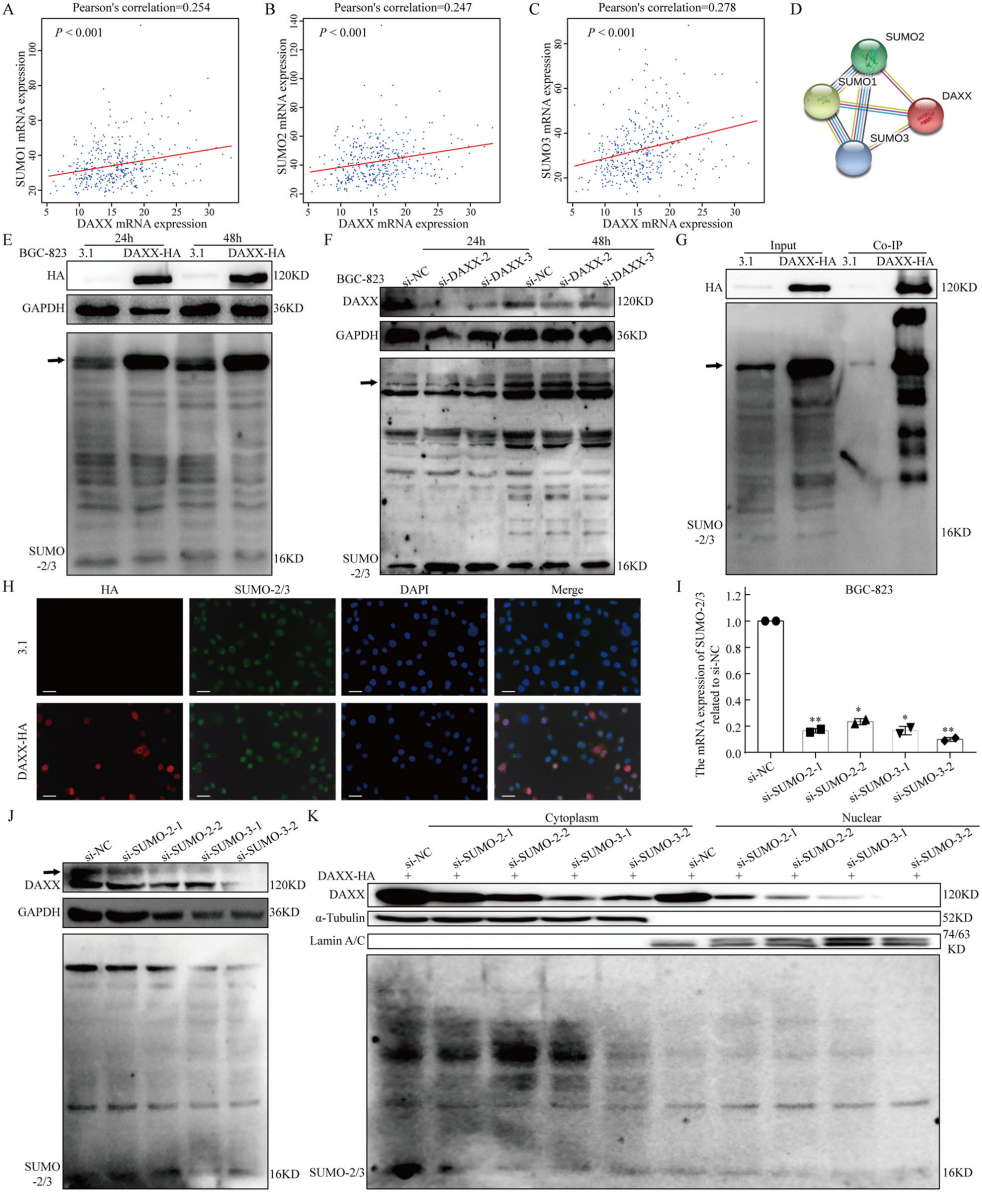
DAXX interacts with SUMO-2/3 and is transported from the cytoplasm to the nucleus. **a**–**c** The expression of DAXX was positively correlated with SUMO-1 and SUMO-2/3 using GEPIA. **d** The STRING database showed that DAXX interact with SUMO-1, 2, 3. **e**, **f** Western blot analysis of DAXX and SUMO-2/3 on BGC-823 transfected with DAXX-HA and si-DAXX. The arrow indicated SUMOylation DAXX. **g**, **h** DAXX interact with SUMO-2/3 directly using co-IP and IF assay. (Scale bar = 10 μm). **i** The SUMO-2/3 mRNA expression was significantly decreased after transfected with si-SUMO-2/3 using qRT-PCR. **j** The DAXX expression was decreased on BGC-823 transfected with si-SUMO-2/3. **k** The DAXX expression in the cytoplasm and nucleus was decreased on BGC-823 transfected with DAXX-HA and si-SUMO-2/3, especially in the nucleus. *P < 0.05, **P < 0.01, and ***P < 0.001.

To further investigate the underlying mechanisms, we examined whether the interaction between SUMO-2/3 and DAXX, and subsequent changes in its subcellular localization may affect the prognosis of patients with GC. Correlation analysis based on GEPIA analysis and the STRING database revealed a positive correlation between DAXX and SUMO-1, 2, 3 (Fig. 4a–d). Meanwhile, we observed changes in SUMO-2/3 expression and SUMOylation levels after DAXX overexpression or knockdown. And western blotting showed that DAXX displayed significant SUMO-2/3 modification (Fig. 4e, f and Supplementary Fig. 2C, D). Co-IP and IF assays clarified that DAXX was a substrate of and directly interacted with SUMO-2/3 (Fig. 4g, h).

Then, we silenced SUMO-2/3 using si-SUMO-2 and si-SUMO-3 (Fig. 4i). Next, we clarified the role of SUMO-2/3 in the subcellular localization of DAXX when SUMO-2/3 expression was knocked down. Overall DAXX expression decreased after knocking down SUMO-2/3 expression (Fig. 4j and Supplementary Fig. 2E); however, when DAXX was overexpressed and SUMO-2/3 was knocked down simultaneously, the expression of DAXX in the cytoplasm and nucleus tended to decrease, with its expression in the nucleus decreasing significantly (Fig. 4k and Supplementary Fig. 2F). Taken together, these results indicate that SUMO-2/3 can not only regulate the subcellular localization and distribution of DAXX, but also enhance its stability.

### RanBP2/RanGAP1 may act as SUMO E3 ligases to promote DAXX SUMOylation

**Fig. 5:**
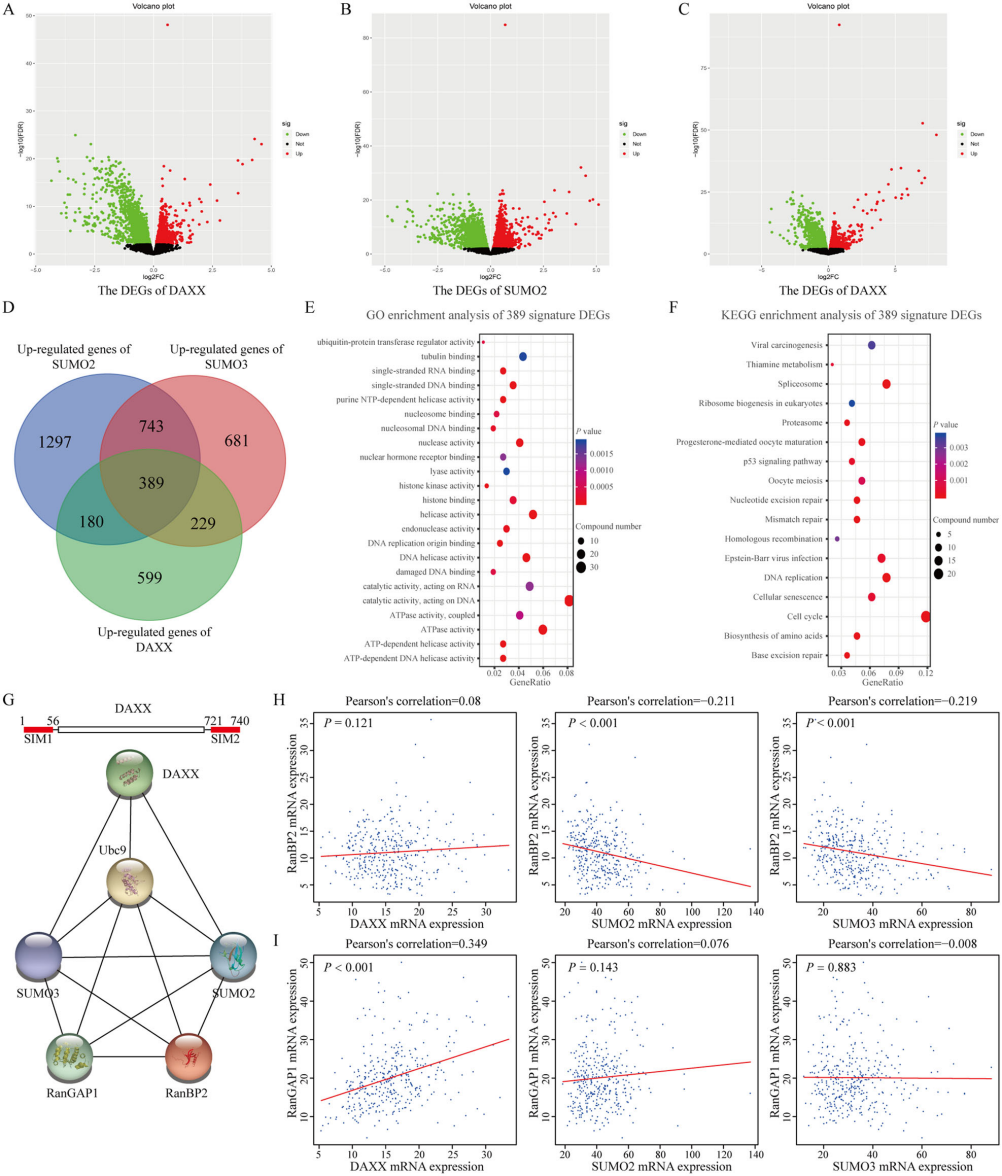
RanBP2/RanGAP1 complex may act as E3 involved in DAXX SUMOylation. **a**–**c** Volcano plot of differentially expressed genes (DEGs) of DAXX, SUMO-2, and SUMO-3. Red spots represent upregulated DEGs and green spots represent downregulated DEGs. **d** Venn diagram illustrating the number of upregulated DEGs in three groups. **e**, **f** Functional enrichment analysis of 389 signature DEGs. **g** STRING database analysis of DAXX, SUMO-2/3, RanBP2, and RanGAP1. **h**, **i** Correlations among DAXX, SUMO-2/3, RanBP2, and RanGAP1.

For substrate SUMOylation, E3 plays an important role in the assembly of a complex consisting of Ubc9 (an E2 ligase), E3, and substrate. Therefore, we next investigated who serves as an E3 in the SUMOylation process of DAXX using bioinformatics. We analyzed DEGs caused by overexpression of DAXX, SUMO-2, and SUMO-3 (Fig. 5a–c). A total of 389 most related genes was obtained from a Venn diagram (Fig. 5d). Functional enrichment analysis was conducted showing that these DEGs were involved in ubiquitin-protein transferase regulator activity (Fig. 5e, f). Then, combining three reported SUMO E3 families, STRING database (Fig. 5g) and correlation analysis (Fig. 5h, i) showed that RanBP2/RanGAP1 complex may act as SUMO E3 in DAXX SUMOylation and nuclear-plasma transport. Taken together, RanBP2/RanGAP1 complex may assemble Ubc9 and DAXX to form SUMOylation complexes and mediate DAXX SUMOylation as potential E3 ubiquitin ligases.

## Discussion

Emerging evidence has suggested that DAXX is a key oncogene in tumorigenesis. Our previous study found that patients with GC often display a higher DAXX NCR^16^; however, the underlying mechanisms of DAXX remains unknown. In this study, for the first time, we demonstrated that cDAXX and nDAXX expression have opposite effects on the survival prognosis of patients with GC. Interestingly, increasing the expression of cDAXX primarily inhibits GC cell proliferation and promotes their migration and invasion, whereas knocking down DAXX reduces nDAXX expression and inhibits GC cell proliferation, migration, and invasion. Moreover, we found that DAXX interacts with SUMO-2/3 in GC, affecting its subcellular localization to exert different biological effects in vitro, which is a novel yet interesting discovery.

DAXX is highly expressed in human organs, particularly various tumor tissues. A previous study used IHC to show that DAXX is expressed in 71.7% of esophageal cancer tissues and is associated with lymph node metastasis and advanced tumor stage^23^. Mahmud and Liao^24^ analyzed DAXX mRNA expression in different cancers and normal tissues using the TCGA database and other published clinical cancer sample data sets, including breast, colon, and liver cancers, finding that DAXX expression was significantly higher in tumor tissues than in normal tissues. In further studies of primary and metastatic tissues, they revealed that DAXX expression was higher in various cancer metastases than in the corresponding primary tumors^24^. Our previous studies^16^ also indicated that increased DAXX NCR was associated with poor clinical outcomes in patients with GC. However, little research has been carried out on the relationship between the subcellular localization of DAXX and the clinical features of GC. In this study, we found that total DAXX expression was higher in tumor tissues from the TCGA dataset than in normal tissues. Then, we examined DAXX expression in 323 GC tissues by IHC. Interestingly, cDAXX and nDAXX expression had opposite effects on the clinical features and survival prognosis of patients with GC. cDAXX expression was associated with lymph node metastasis, Lauren type, and better overall survival in GC patients (*P* < 0.01), whereas high nDAXX expression often suggests a higher rate of recurrence and a poorer survival rate (*P* < 0.01).

DAXX has been shown to play a role in complex biological functions. The protein was originally found to be a Fas-mediated pro-apoptotic protein in HeLa cells, involved in the caspase-independent pathway by activating apoptosis signaling regulator kinase 1 and c-Jun NH2 terminal kinase^4^. Recently, there is increasing evidence that DAXX can act as a pro- or anti-apoptotic factor in cell survival and apoptosis depending on its subcellular localization. In addition, DAXX is critical for cancer progression, metastasis, and drug sensitivity^8,25,26^. For instance, in some types of cancer, DAXX can act as a transcriptional co-repressor to inhibit oncogene expression or promote Fas-mediated cell death. Lin et al.^27^ found that hypoxia-inducible factor (HIF)-1α can promote cancer invasion under hypoxic conditions by down-regulating DAXX expression. Moreover, studies have reported that DAXX can reduce T-cell receptor (TCR) complex-induced cell proliferation and promote Fas-mediated cell death^28^. In contrast, DAXX was found to be closely related to cell proliferation, metastasis, and the development of drug resistance in ovarian cancer^8^, while Liu et al.^29^ found that DAXX promotes the growth, proliferation, and migration of ovarian cancer ascites cells in vivo by activating ERK signaling pathways. Interestingly, glioma-related studies^30^ have demonstrated that cancers can survive forever and escape cellular senescence and apoptosis. However, the relationship between nuclear and cytoplasmic DAXX expression, and the malignant phenotype of GC cells remains unclear.

In our study, we found that there was no significant difference in the mRNA levels of DAXX between different cell lines, but the protein levels of DAXX have changed. This phenomenon may be caused by the post-transcriptional modifications of DAXX. There are three main post-transcriptional modifications in DAXX, including phosphorylation, ubiquitination, and SUMO modification. Our results have showed that SUMO modification can promote the stability of DAXX protein, thereby regulating many cell activities (Fig. 4). In contrast, ubiquitination modification will induce the degradation of DAXX. Therefore, the expression level of DAXX at the protein level depends on the interaction and regulation of the two post-transcriptional modification. This may explain the changes in protein levels to a certain extent. Then, we performed nuclear/cytoplasmic protein separation assays on GC cells and found that DAXX overexpression mainly caused a significant increase cDAXX expression, whereas silencing DAXX significantly decreased nDAXX expression. Therefore, we thought that DAXX overexpression can significantly inhibit GC cell proliferation but promote their apoptosis, migration, and invasion. Further studies showed that knocking down DAXX can significantly inhibit the proliferation, anti-apoptotic effects, migration, and invasion of GC cells. In addition, we speculated that DAXX regulates cell migration and invasion mainly through its expression in the nucleus, which needs further verification in subsequent experiments. This in vitro result was also consistent with our IHC results, revealing that DAXX has certain carcinogenic properties and may be a potential therapeutic target.

**Fig. 6:**
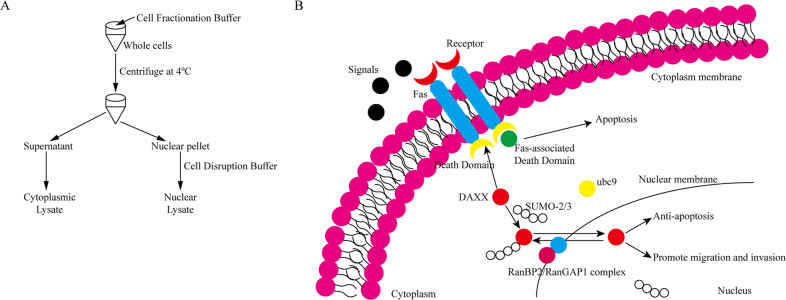
A schematic illustration of the role of DAXX and SUMO-2/3. **a** An illustration of the separation of nuclear and cytoplasmic proteins. **b** The model of SUMO-2/3-mediated DAXX nuclear-cytoplasmic transport.

DAXX is generally found in the nucleus, cytoplasm, and some other subnuclear structures, and interacts with different proteins, with its function depending to a large extent on its subcellular localization, which is closely related to SUMOylation^15,31^. Multiple studies have shown that SUMO can directly interact with DAXX or conjugate with DAXX-related proteins to indirectly alter the subcellular localization of DAXX^15^. It has been reported that Ubc9, an E2-conjugated enzyme required for SUMOylation, interacts with DAXX and that subsequent changes in the subcellular localization of DAXX may contribute to its sensitivity to chemotherapeutic drugs^25^. The majority of studies have focused on the interaction between DAXX and SUMO-1^32–34^. In contrast, SUMO-2/3 quickly binds to the substrate under stress conditions, causing a series of defense responses^35,36^. Moreover, the deSUMOylating enzyme SENP3 that specifically targets SUMO2/3 was identified as an oxidative stress-responsive molecule^37–39^. SUMO-2/3 modifications can also regulate molecular localization; therefore, in this study we explored whether SUMO-2/3 can affect the subcellular localization of DAXX. Using the TCGA database, we found a positive correlation between DAXX and SUMO-2/3 mRNA transcription levels, confirming a direct interaction between DAXX and SUMO-2/3 through IF and co-IP experiments. Moreover, we showed that DAXX overexpression can significantly promote its SUMOylation, yet its SUMOylation was unaffected by silencing DAXX. Therefore, we hypothesized that during tumorigenesis and development the human body produces intense stress and defense responses that promote the conjugation between SUMO-2/3 and DAXX, causing DAXX to be transferred from the cytoplasm to the nucleus and increasing the malignancy of the tumor (Fig. 6).

We silenced SUMO-2/3 expression to further verify whether SUMO-2/3 mediates the nuclear-cytoplasmic transport of DAXX, finding that silencing SUMO-2/3 expression significantly decreased overall DAXX expression and also SUMOylated DAXX expression. Moreover, overexpressing DAXX and silencing SUMO-2/3 simultaneously caused DAXX to accumulate in the cytoplasm and significantly decreased its expression in the nucleus. These results indicate that SUMO-2/3 is a major factor that mediates the nuclear-cytoplasmic transport of DAXX and, to some extent, leads to its degradation, which may be a direction for research in future studies.

SUMOylation has three key steps, each of which depending on a specific enzyme. E3 ubiquitin ligase interacts with E2-Ubc9 and a specific substrate to form a complex, and then mediates the transfer of SUMO to the substrate. However, there were no clear E3 been identified in DAXX SUMOylation. In this study, we compared and analyzed the DEGs of DAXX and SUMO-2/3 with three reported SUMO E3 families. Analysis showed that RanBP2 may be a potential E3. In addition, previous study has confirmed that RanBP2 was not only a SUMO E3, but also an essential protein for nuclear-plasma transport. RanBP2 can form a complex with RanGAP1, which can effectively regulate the nuclear-plasmon transport of other proteins. However, their specific binding methods and locations need to be verified by further experiments.

In summary, we demonstrated that the subcellular localization of DAXX plays a key role in the clinical features and survival prognosis of GC patients as well as the biological functions of GC cells. SUMO-2/3 can affect the response of GC cells to proliferation and invasion by altering the subcellular localization of DAXX. The accumulation of nDAXX is often associated with a poorer prognosis for patients with GC. Therefore, DAXX may be a novel molecular target for predicting and treating GC. Detailed mechanisms via which the interaction between DAXX and SUMO-2/3 mediates transport and degradation should be explored in future studies.

## Supplementary information

Supplementary Figure 1

Supplementary Figure 2
