# Supplementary figures and images for: Opposing biological functions of the cytoplasm and nucleus DAXX modified by SUMO-2/3 in gastric cancer
Source: Cell Death Dis. 2020 Jul 8;11(7):514. doi: 10.1038/s41419-020-2718-3 (PMC7343808; doi:10.1038/s41419-020-2718-3)

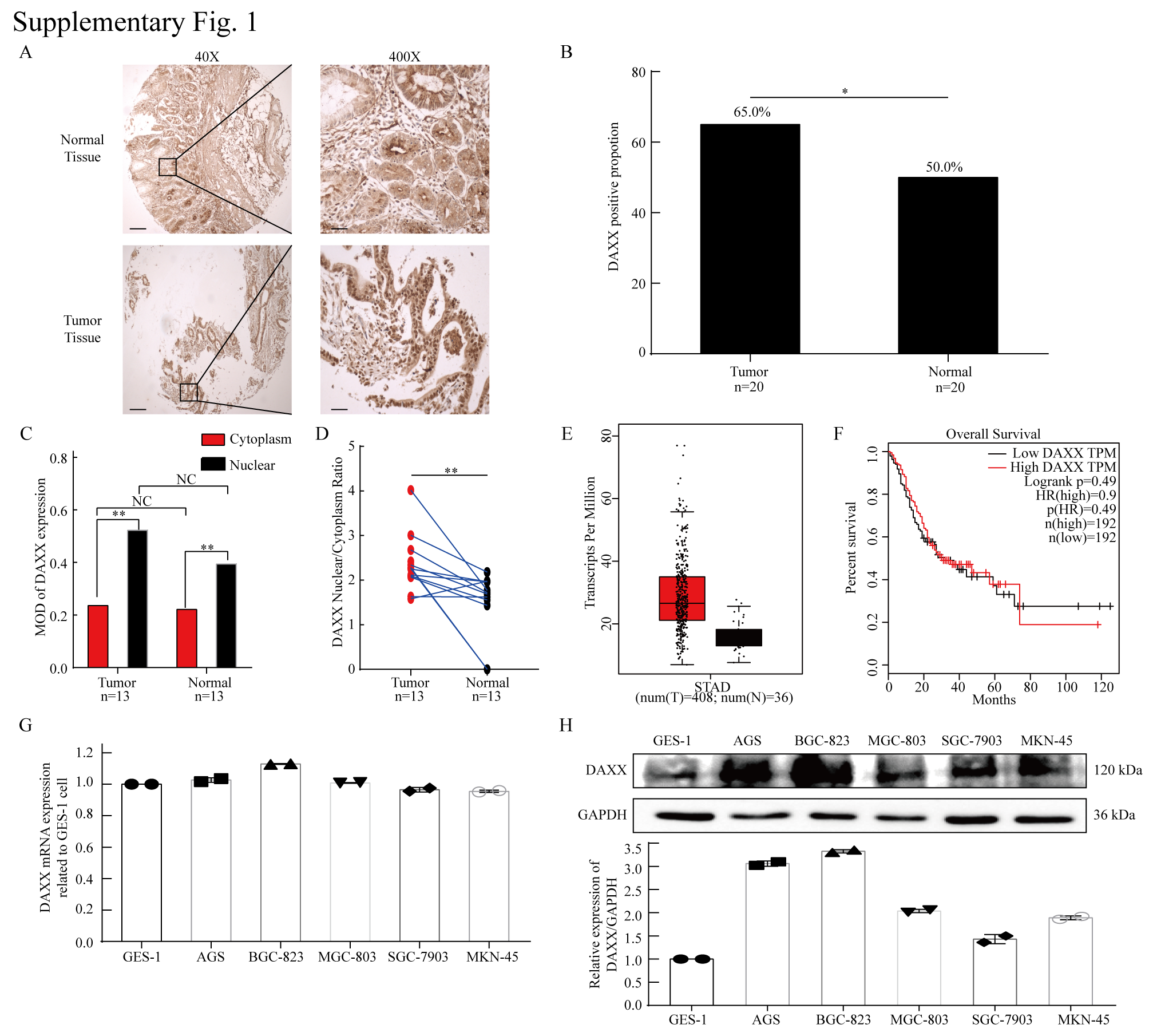

Supplement: Supplementary file 1 — Supplementary Figure 1 [file 41419_2020_2718_MOESM1_ESM.tif]

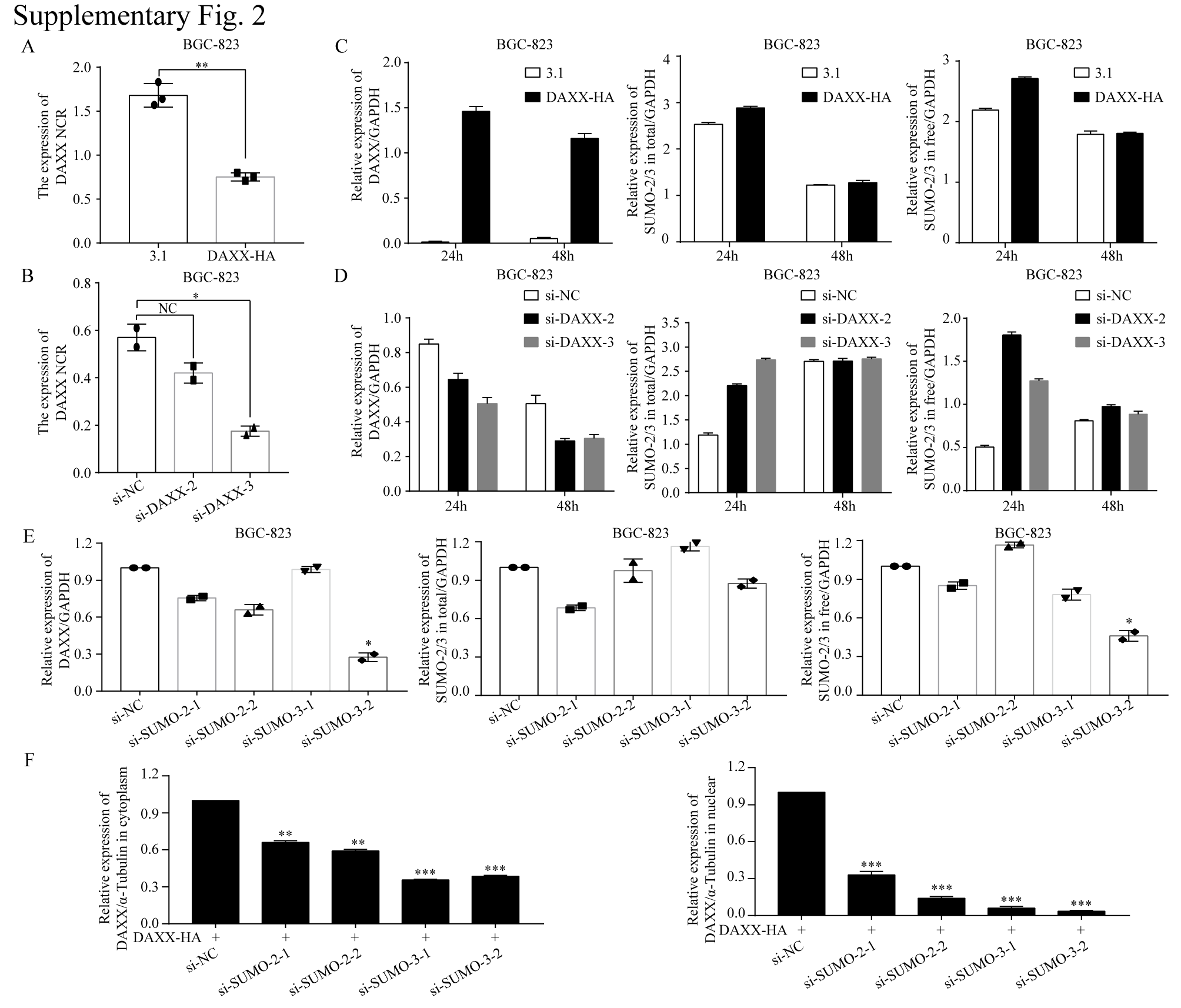

Supplement: Supplementary file 2 — Supplementary Figure 2 [file 41419_2020_2718_MOESM2_ESM.tif]
